# Computational Study of Terahertz-Induced Phase Transitions at Molecular Interfaces

**DOI:** 10.34133/research.1350

**Published:** 2026-07-28

**Authors:** Chenzhi Tang, Dexing Shen, Zhi Du, Yong He, Yingying Sun, Ze Wang, Yuanyuan He, Xingang Ren, Kaijie Wu

**Affiliations:** ^1^ School of Electronic and Information Engineering, Anhui University, Hefei 230601, China.; ^2^ School of Electronics, Peking University, Beijing 100871, China.; ^3^ School of Safety Engineering, University of Emergency Management, Hebei 065201, China.; ^4^ Information Materials and Intelligent Sensing Laboratory of Anhui Province, Anhui University, Hefei 230601, China.

## Abstract

Structural phase transitions driven by atomic rearrangements at the interaction interfaces of biological macromolecules, particularly proteins, are fundamental to regulating critical physiological processes, making their effective modulation key to developing targeted therapeutics. Existing induction strategies encounter substantial limitations: Chemical methods have difficulty accessing shallow binding pockets and can lead to invasive, irreversible modifications, while common visible and near-infrared modulation technologies are hindered by side effects induced by exogenous agents and restricted deep-tissue penetration. Here, using molecular dynamics simulations of the programmed cell death protein 1/programmed cell death 1 ligand 2 complex, we find terahertz light (40.2 THz) to be a reversible, nonthermal, and noninvasive physical induction modality to drive a directed structural phase transition. Energetic analyses reveal that the terahertz field markedly decreases the binding affinity by more than 3-fold and reduces the free energy barrier by nearly 3-fold. Fundamentally, our research demonstrates that this directed decoupling process originates from the coherent resonance of certain key interfacial residues. Collectively, our findings propose a universal physical strategy to modulate macromolecular interfaces, providing an essential computational framework to guide future experimental validations in noninvasive tumor immunotherapy.

## Introduction

The dynamic interactions of biological macromolecules, particularly the specific recognition and binding at protein–protein interaction interfaces, constitute the fundamental physicochemical basis for executing complex functions within living systems, driving ubiquitous physiological events such as cellular signal transduction, enzymatic cascades, and the regulation of gene transcription [1,2]. A prominent example is the programmed cell death protein 1/programmed cell death 1 ligand 2 (PD-1/PD-L2) immune checkpoint complex, whose interfacial binding mediates tumor immune evasion and represents a paramount target in cancer immunotherapy [3]. Crucially, such interfacial binding depends not merely on static physical contact but profoundly on the intricate dynamics of conformational evolution, as these binding interfaces function as inherently dynamic systems maintained by weak intermolecular forces undergoing continuous stochastic fluctuations on time scales ranging from femtoseconds to microseconds [4,5]. This intrinsic dynamic nature enables the binding interface to undergo induced structural phase transitions, which ultimately trigger functional state switching [6,7]. Consequently, inducing rational structural phase transitions to achieve steady-state switching of biological signals has emerged as a central challenge in synthetic biology, protein engineering, and modern drug discovery.

Inducing interfacial phase transitions typically requires active external stimuli, currently dominated by traditional biochemical and genetic interventions. Monoclonal antibodies remain the mainstay of clinical therapy due to their exceptional specificity and ability to achieve precise tumor targeting [8,9]. For example, antibodies targeting PD-1 effectively disrupt the PD-1/PD-L2 binding interface to restore T cell cytotoxicity against malignant cells [10]. Although these agents provide potent blockade of functional coupling, their large molecular weight and steric effects often constrain their capacity for reversible modulation. In contrast, small-molecule inhibitors and peptidomimetics offer the advantage of precisely targeting critical contact sites to competitively block interfacial binding [11,12]. However, the broad and flat topography of biomolecular interaction interfaces, characterized by shallow binding pockets, makes it difficult for these traditional drugs to achieve sufficient access and affinity, often leading to persistent challenges of off-target toxicity. Alternatively, while common visible and near-infrared modulation technologies, such as optogenetics, have demonstrated remarkable success in providing spatiotemporal control over interfacial functions [13,14], they are fundamentally hindered by side effects induced by exogenous agents and restricted deep-tissue penetration. Consequently, although these existing methods provide powerful tools for intervening at interfaces, their inherent limitations necessitate the exploration of universal, reversible, and noninvasive physical strategies.

Terahertz (THz) light provides a possibility to circumvent these challenges. Unlike ionizing radiation or high-energy lasers, this THz light specifically resonates with collective hydrogen bond vibrations and backbone dynamics, enabling precise intervention in biological macromolecules while preserving their structural integrity [15–19]. Chang’s team. [20] proposed that THz light can exert substantial modulatory effects on specific biological tissues or cells, elucidating the nonthermal resonance mechanisms between these electromagnetic (EM) fields and biomolecules. For instance, their team demonstrated that THz EM stimuli resonate with purine DNA vibrations to break intermolecular bonds and accelerate the DNA unwinding process [21]. Similarly, they also discovered that a specific THz field can actively alleviate comorbidities by reducing the interfacial binding capacity between nanostructured molecules and receptors [22]. Furthermore, they demonstrated that THz EM stimuli nonthermally regulate neuronal signaling by resonating with carbonyl bonds in potassium channels [23]. Additionally, they applied exogenous gene-free THz modulation to activate cortical neurons in vivo and markedly accelerate associative learning in mice [24]. Recently, Wang’s team [25] also verified the feasibility of utilizing transcranial high-frequency THz stimulation as a noninvasive approach to effectively alleviate complex behavioral disorders in animal models. Regarding the theoretical interpretation of resonance between THz light and biomolecules, Zhu’s team [26] proposed that specific-frequency THz irradiation overcomes the free energy barriers of distinct isomers to achieve controllable molecular isomerization. Moreover, they introduced a theoretical design utilizing a specific-frequency THz field to resonate with vicinal subnanoscale water layers, achieving remote, controllable, and reversible modulation of surface wettability by altering the local hydrogen-bonding network [27]. Given that the dynamic phase transitions of biomolecular interfaces are intrinsically governed by the same complex hydrogen-bonding networks and local conformational energy barriers investigated in these preceding studies, THz light holds immense potential as a reversible, low-damage optical intervention modality at biomolecular complex interfaces.

**Fig. 1.**
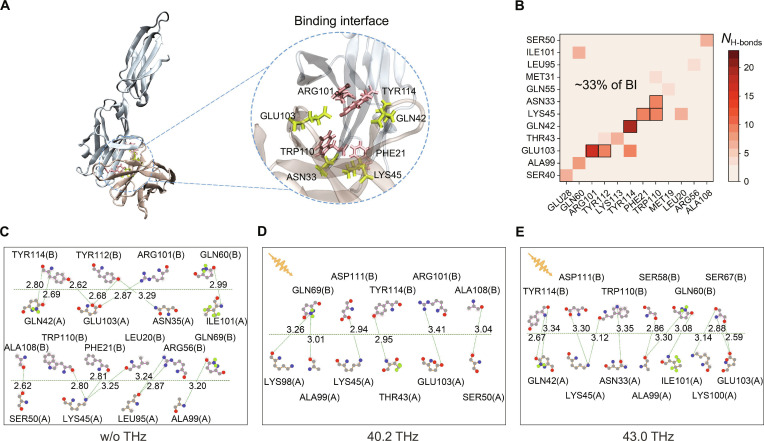
Structural basis of the programmed cell death protein 1/programmed cell death 1 ligand 2 (PD-1/PD-L2) binding interface and terahertz (THz)-induced interfacial structural phase transition. (A) Molecular dynamics (MD) equilibrium conformation of the PD-1/PD-L2 complex and a close-up view of the binding interface. PD-1 and PD-L2 are colored in light brown and light blue, respectively. Interface residues originating from PD-1 are shown in yellow, while those originating from PD-L2 are shown in pink. (B) Key interfacial residues sustaining interface stability and their total number of hydrogen bonds across 10 representative frames. (C to E) Visualization of the topological structure evolution of the interfacial H-bond network under the applied THz field. Atoms are colored as follows: carbon (gray), nitrogen (blue), oxygen (red), and other atoms (green). Green lines denote H-bond connections between residues.

In this study, we aim to elucidate the feasibility of utilizing THz light to drive structural phase transitions at biological macromolecular interfaces. To validate this THz field induction strategy, we employed the PD-1/PD-L2 complex as our theoretical model (Fig. 1A). Beyond the profound clinical importance, this characteristically flat interface lacking deep binding pockets serves as a highly representative paradigm for challenging protein–protein interaction targets. Based on an all-atom model, we demonstrate that a frequency-specific (e.g., 40.2 THz) THz EM stimulus can drive controllable structural phase transitions at the interface. Specifically, these THz EM stimuli trigger precise and reversible coherent resonance within key interfacial residues to effectively inhibit PD-1/PD-L2 binding. These findings demonstrate that THz light can precisely induce structural phase transitions at biomolecular interfaces, manifesting as a structural rearrangement of interfacial connections from a stable dense state to a sparse state and providing a robust theoretical foundation for the rational design of THz-based immunotherapies.

## Results and Discussion

### Identification of key interface residues

By analyzing 10 representative conformations extracted from the equilibrated all-atom molecular dynamics (MD) trajectories, we dissected the intermolecular interaction network between the interfacial residues of PD-1 and PD-L2 using the LIGPLOT+ software. Figure 1A clearly illustrates the spatial distribution of core residues within these interfacial regions, revealing a dense interaction network that underpins the physical connectivity of the interface structure. Given the pivotal role of H-bond networks in mediating protein interface recognition and maintaining conformational flexibility, we quantified the total number of H-bonds ($N_{H-\text{bonds}}$) formed by each residue across these 10 frames as a metric for interfacial coupling strength. As shown in Fig. 1B, statistical analysis identified 8 key residues—including ASN33, LYS45, GLN42, GLU103, ARG101, TYR114, PHE21, and TRP110—as core anchoring residues, defined by $N_{H-\text{bonds}}\geq 10$. Such high values physically signify that these “hotspot” residues form either highly persistent H-bonds or multiple interconnected H-bond networks. They represent approximately 33% of the total residues at the binding interface, constituting the physical pillars that enable the complex to resist thermal fluctuations and maintain a tight conformation.

We found that THz light exhibits pronounced frequency selectivity in driving interfacial structural evolution and state transitions. Analysis of the molecular interfacial interaction network clearly captured this dynamic transition process triggered by specific THz field frequencies (Fig. 1C to E). In the absence of an external field (Fig. 1C), the interface presented a dense and ordered H-bonds connectivity network, ensuring the tight engagement of PD-1 and PD-L2. In contrast, under the influence of 40.2-THz excitation (Fig. 1D), the interfacial network underwent the most profound structural phase transition: The originally dense H-bonds connectivity evolved into a sparse and loose topological distribution. This topological evolution signifies a phase transition of the interface from a tightly bound state to a loosely dissociated state. This sparsification effect was also evident in the 43.0-THz group (Fig. 1E), where the interface exhibited a certain degree of structural relaxation. In the 36.6-THz group (Fig. S3), although the overall topology maintained a density highly similar to that of the field-free group, subtle structural perturbations were still captured: We observed a mild increase in the average bond length of certain interfacial H-bonds and a slight attenuation trend in the total number of H-bonds. This frequency-dependent structural response demonstrates that THz light can act as a noninvasive physical modulator, inducing varying degrees of interfacial structural phase transitions by tuning the excitation frequency, thereby precisely and controllably sparsifying the critical physical connections that maintain biological macromolecular complexes.

### Frequency-selective resonance and vibrational modes

Given that the microscopic essence of the interfacial structural phase transition lies in the dynamic conformational adjustments of key interfacial-residue side chains, we investigated the THz frequency (*v*) response characteristics of the PD-1/PD-L2 interface. To avoid severe EM shielding caused by the strong absorption of environmental water (5 to 32 THz), while bypassing high-frequency intramolecular covalent bond vibrations (>50 THz) and low-frequency, low-absorption regions (0 to 5 THz), we selected 32 to 44 THz as the optimal frequency window (Fig. S4). Within this frequency regime, the applied THz field can achieve strong resonant coupling with specific localized vibrational modes within the highly polar noncovalent interfacial network, thereby driving large-scale side-chain torsions and collective conformational remodeling. The PD-1/PD-L2 interface comprises a complex mixture of nonpolar and polar groups, where key residues exhibit marked asymmetry and possess permanent dipole moments. Consequently, the torsion and oscillation of these residue side chains induce drastic fluctuations in their dipole moments, generating characteristic absorption fingerprints in the THz region. To identify the specific vibrational modes of these core anchoring sites and directly correlate them with THz absorption, we focused our spectral analyses specifically on these key residues, combining MD simulations with density functional theory (DFT).

**Fig. 2.**
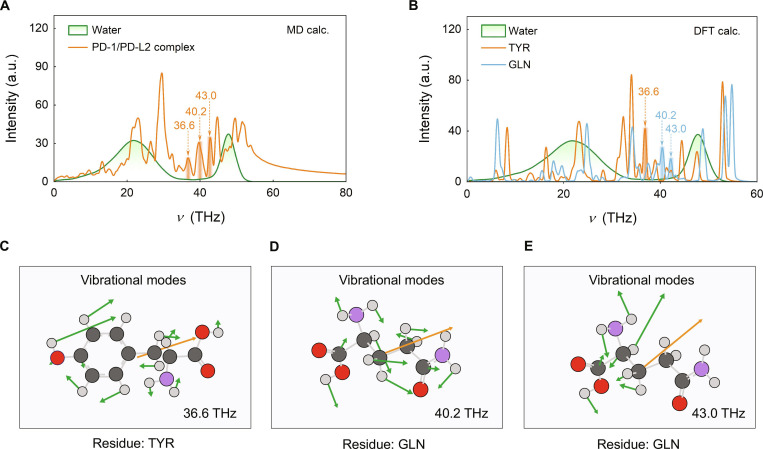
Frequency-selective resonance characteristics and molecular vibrational modes of the PD-1/PD-L2 binding interface. (A) Molecular dynamics (MD) derived absorption spectra of the core interfacial residues within the terahertz (THz) frequency range. (B) Density functional theory (DFT) calculated absorption spectra of glutamine (GLN) and tyrosine (TYR). (C to E) Visualization of the quantum mechanical eigen-vibrational modes for key residues at characteristic frequencies. Atoms are colored as follows: carbon (dark gray), hydrogen (light gray), nitrogen (purple), and oxygen (red). Vector arrows indicate the displacement amplitude and direction of atomic vibrations driven by specific resonant frequencies.

MD simulation results (Fig. 2A) reveal that, after excluding the background interference of solvent water molecules, these key anchoring residues exhibit 3 prominent resonant absorption peaks at 36.6, 40.2, and 43.0 THz. These discrete absorption features not only correspond to the intrinsic collective vibrational modes of these specific residues but also reveal the existence of highly localized resonant coupling windows at the binding interface. When the incident field frequency precisely matches these eigenmodes, the EM energy is efficiently absorbed by these core residues via coherent coupling and converted into kinetic energy to overcome local potential barriers. This nonequilibrium dynamic effect, driven by resonance, provides a physical basis for the precise modulation of interfacial binding affinity.

Further elucidation of the microscopic processes based on DFT confirms that this frequency selectivity stems from the fingerprint-like capture and mechanical conversion of the EM energy by specific residue side chains. Computational results indicate that GLN exhibits strong resonant absorption near 40.2 and 43.0 THz, while TYR shows similar behavior in the vicinity of 36.6 THz (Fig. 2B). Although damping effects introduced by the explicit solvent environment in all-atom MD simulations and spectral broadening caused by thermal fluctuations result in a certain physical frequency shift between classical MD simulations and quantum mechanical DFT calculations, both methods still exhibit a high degree of spectral overlap in the overall envelope of the characteristic frequency bands. This consistency between the classical statistical and quantum mechanical levels strongly validates our identification of the resonance centers. Corroborating this, our extended analysis of other key interface residues (Fig. S5) also shows that certain residues exhibit matching absorption peaks within these characteristic frequency bands. This multidimensional chain of spectral evidence confirms that these 3 frequencies serve as critical transmission channels for coupling the resonant EM energy to the biomolecular interface, rather than merely acting as characteristic fingerprints. Furthermore, resonance absorption by isolated residues does not guarantee macroscopic dissociation. For instance, exciting individual hotspots (e.g., ARG at 32.0 THz, GLU at 34.5/38.0 THz, and TYR at 36.6 THz) only marginally attenuates binding energy without triggering interfacial decoupling (Fig. S6). Because the protein interface operates as a synergistic mechanical network, localized vibrations are negated by the spatial cancellation of atomic displacement vectors—such as the opposing motions of GLU and GLN under 38-THz excitation (Fig. S7). Effective macroscopic phase transitions require the external field to match collective superposition modes to generate a net resultant force. Additionally, specifically at 32.0 THz, surrounding water absorption causes energy dissipation, further preventing effective energy transfer to the binding interface.

Building on this spectral matching, resonance vector analysis visualizes the vibrational modes associated with this energy coupling. Specifically, the 36.6-THz mode excites the side-chain deformation of TYR, facilitating the relaxation of local hydrophobic contacts (Fig. 2C). At 40.2 THz, the resonance drives collective vibrational modes within the GLN residue, where energy couples into both the side chain and backbone to generate a coordinated rocking motion (Fig. 2D). Furthermore, the 43.0-THz mode is characterized by synchronized vibrations within the GLN residue, which enhances local structural fluctuations and perturbs the interfacial steady state (Fig. 2E). Conversely, at nonresonant frequencies, the vibrational modes exhibit conflicting atomic displacement vectors that neutralize the overall dipole moment change (e.g., TYR at 40.2 THz; Fig. S8), thereby resulting in lower absorption. Collectively, these nondestructive vibrations provide the kinetic driving force for interfacial sparsification, demonstrating the potential of THz light as a precise, reversible physical regulator for the functional remodeling of biomolecular interfaces at the atomic scale.

### Thermodynamic steady-state transition of the binding energy

**Fig. 3.**
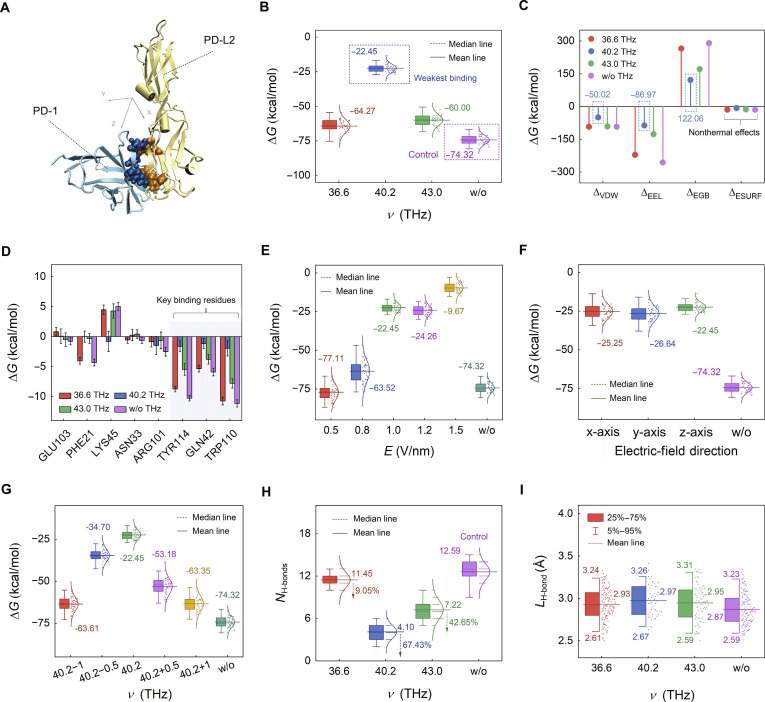
Comprehensive dynamic analysis and H-bond network reconfiguration driving the PD-1/PD-L2 interfacial structural phase transition. (A) Visualization of the PD-1/PD-L2 binding interface. (B) Comparison of binding energies under varying terahertz (THz) field frequencies. (C) Changes in the contributions of binding energy components. (D) Changes in the binding energy contributions of key interfacial residues. (E) Changes in the binding energy of the complex under varying 40.2-THz electromagnetic (EM) field strengths. (F) Changes in the binding energy of the complex under 40.2-THz EM fields applied along different spatial axes. (G) Changes in the binding energy of the complex under THz EM excitations at frequencies adjacent to 40.2 THz. (H) Changes in the number of H-bonds within the PD-1/PD-L2 complex. (I) Changes in H-bond lengths within the PD-1/PD-L2 complex.

To elucidate the physical basis by which this frequency selectivity drives the controllable modulation of interfacial binding affinity, we constructed an interaction network model of the PD-1/PD-L2 binding interface (Fig. 3A) and further analyzed its energy evolution characteristics from a thermodynamic perspective. Figure 3B reveals the substantial remodeling effect of the applied THz field on the binding energy (ΔG) of the PD-1/PD-L2 complex. Specifically, THz excitation at 36.6 and 43.0 THz shifted the binding energy from the field-free equilibrium state of −74.32 kcal/mol to −64.27 and −60.00 kcal/mol, respectively. This directly reflects the relaxation of noncovalent affinity and the shallowing of the interfacial potential well. In contrast, the induction effect at 40.2 THz was more pronounced, with the complex binding energy shifting markedly to −22.45 kcal/mol. This substantial energy transition marks the interface being driven from a low-energy tightly bound state to a high-energy sparse state.

Energy component analysis further revealed the driving factors of this state transition (Fig. 3C). First, the absolute values of both electrostatic interaction energy ($\Delta_{\mathrm{EEL}}$) and polar solvation energy ($\Delta_{\mathrm{EGB}}$) exhibited attenuation across all THz-modulated groups. This universal phenomenon indicates that the THz EM field relaxed the interfacial compactness and promoted water molecule penetration, thereby masking the originally strong electrostatic attraction through dielectric screening effects and lowering the desolvation energy barrier, effectively driving the transition of the original dry interface into a solvated state. On this basis, the 40.2-THz group displayed a unique feature: Its van der Waals interaction energy ($\Delta_{\mathrm{VDW}}$) was drastically weakened, shifting to −50.02 kcal/mol. This critical feature implies that this frequency induced deep conformational adjustments and a relaxation of atomic packing density, resulting in a conformational mismatch in the originally precise shape complementarity of the interface. Notably, the nonpolar solvation energy ($\Delta_{\text{ESURF}}$) remained highly stable and close to zero across all groups, effectively ruling out the possibility of pure thermal effects and providing definitive thermodynamic evidence for the dominance of the coherent resonance nonthermal regulatory process. Finally, we profiled the contribution distribution of different residues to the total binding energy (Fig. 3D), focusing on the 3 core residues with the largest contributions: TYR114, GLN42, and TRP110. The data show that under resonant THz stimulation, the binding energy contributions of these 3 key residues all exhibited marked attenuation. This phenomenon strongly confirms that the applied THz field does not induce a global, nonspecific excitation of the entire protein, but rather, by precisely weakening the interactions of these key interfacial residues, it effectively loosens the mechanical support network of the interface, thereby driving the interfacial structural phase transition.

To establish the physical picture of this EM-induced dissociation, we evaluated the system’s parameter sensitivity under the principal 40.2-THz resonance. First, the dissociation exhibits a prominent nonlinear threshold effect regarding electric field amplitude (Fig. 3E). Under weak fields (≤0.8 V/nm), the binding energy remained largely stable (slightly decreasing to −63.52 kcal/mol at 0.8 V/nm) due to the complex’s intrinsic structural resilience. However, traversing the 0.8 to 1.0 V/nm threshold plunged the binding energy drastically to −22.45 kcal/mol, defining this range as the minimum effective dose for macroscopic decoupling. Meanwhile, although a 1.5 V/nm field drove nearly complete dissociation, it triggered a massive surge in global solvent-accessible surface area (SASA) (Fig. S9), indicating irreversible thermal denaturation [28]. Furthermore, this dissociation demonstrates remarkable spatial isotropy (Fig. 3F). Orthogonal 40.2-THz field applied along the X, Y, and Z axes uniformly induced comparable binding energy reductions (−25.25, −26.64, and −22.45 kcal/mol, respectively). This axis-independent efficacy stems from the complex 3-dimensional interfacial topology, where the resonant absorption dipole moment acts as a 3-dimensional tensor with substantial components across all spatial axes. Finally, frequency sweeping identified a physical effective bandwidth between 39.7 and 40.2 THz (Fig. 3G), where the incident THz field induced marked structural phase transitions (lowering binding energy from the −74.32 kcal/mol baseline to −34.70 and −22.45 kcal/mol, respectively). Beyond this window (e.g., at 39.2 and 41.2 THz), modulatory efficacy plummeted, with binding energies recovering to −63.61 and −63.35 kcal/mol, aligning with the intrinsic vibrational absorption peak’s coverage. Based on these evaluations, a Z-polarized 1.0 V/nm electric field was established as the standard configuration for subsequent simulations.

Subsequently, we investigated the modulatory effect of THz light on the interfacial H-bonds network from a structural topology perspective. As shown in Fig. 3H, the THz field induced a sparsification of the complex’s interfacial H-bonds network. Statistical analysis indicates that under the excitation of 36.6-, 40.2-, and 43.0-THz EM field, the average number ($N_{H-\text{bonds}}$) of interfacial H-bonds dropped from 12.59 in the control group to 11.45, 4.10, and 7.22, respectively. This reduction in the number of physical connections directly led to a relaxation of interfacial topological constraints and was a major factor driving the transition of the interface toward a sparse state. Beyond the substantial reduction in network density, the physical properties of the remaining H-bonds also changed. We observed that the average H-bonds length ($L_{H-\text{bonds}}$), extracted from static representative frames, generally exhibited an elongation trend, stretching from an initial 2.87 Å to 2.93, 2.97, and 2.95 Å, respectively (Fig. 3I). To exclude sampling randomness, full-trajectory statistical analysis (Fig. S10) further confirmed the consistent physical trend of H-bond elongation. Furthermore, the general increase in the average distance between heavy donor–acceptor atoms (N⋯O, Fig. S11) attenuates intermolecular electrostatic attraction, indicating a transition toward a looser binding mode. Finally, normal mode analysis reveals that this structural degradation stems from the selective excitation of localized vibrational modes in terminal polar bonds (e.g., O–H and N–H). The resulting intense spatial oscillations mechanically stretch or sever these noncovalent linkages (Fig. S12), ultimately driving interfacial sparsification.

### Energy landscape of conformational transition

**Fig. 4.**
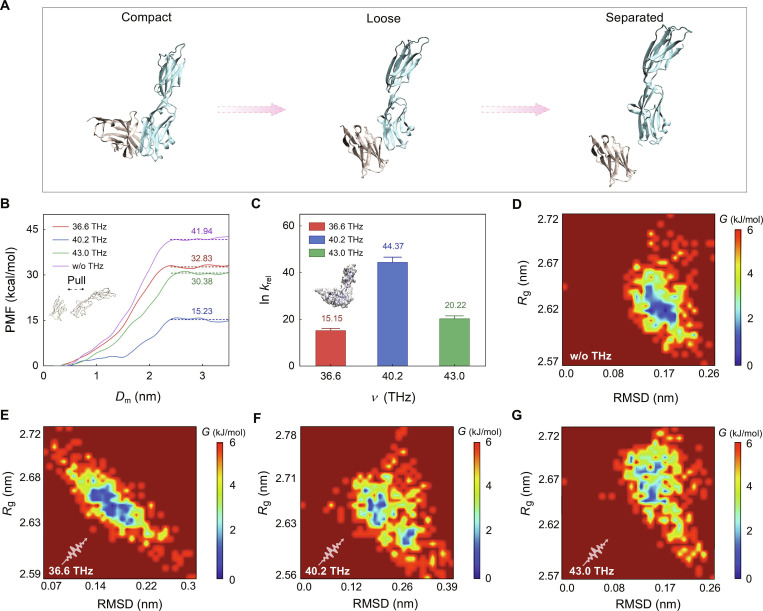
Energy barriers and conformational dynamics of the PD-1/PD-L2 interface under the applied terahertz (THz) field. (A) Schematic of the pulling process used in potential of mean force (PMF) calculations (PD-1, light brown; PD-L2, light blue). (B) Profiles of the PMF along the reaction coordinate. (C) Changes in the natural logarithm of the relative dissociation constant ($\ln k_{\mathrm{rel}}$). (D to G) Free energy landscapes of the PD-1/PD-L2 complex. RMSD, root-mean-square deviation.

Based on the structural analysis of the sparsification of the interfacial microscopic topological network, we further quantified the remodeling effect on the energy landscape along the reaction path (pulling schematic shown in Fig. 4A) via potential of mean force (PMF) calculations. The PMF profiles (Fig. 4B) intuitively depict the energy landscape of the complex along the dissociation pathway ($D_{m}$). In the native equilibrium state without external field interference, the system must overcome a substantial free energy barrier of 41.94 kcal/mol to dissociate. This robust thermodynamic wall restricts conformational degrees of freedom, safeguarding the structural rigidity of the complex under physiological conditions. However, the introduction of the external THz field substantially attenuated this barrier, drastically lowering the energy threshold for conformational transitions. Specifically, under excitation at 36.6, 40.2, and 43.0 THz, the dissociation barriers were reduced to 32.83, 15.23, and 30.38 kcal/mol, respectively. This pronounced attenuation in barrier height reveals the underlying physical basis: The incident THz field, through resonant coupling with core interfacial residues, converts EM energy into local mechanical vibrational energy. This compensates for part of the activation energy required for dissociation, enabling the complex to cross the barrier at a lower energetic cost and achieve a transition from a stable dense state to a sparse state.

To quantify this dynamic modulation effect, we constructed a relative dissociation rate model based on the classical Arrhenius equation. The Arrhenius equation quantitatively describes the exponential relationship between the reaction rate constant (k) and the activation energy barrier [29]:

$$k=A\times e^{-\frac{F}{\mathrm{RT}}}$$ (1)

where *A* is the pre-exponential factor, *F* is the reaction energy barrier (corresponding here to the PMF barrier), *R* is the ideal gas constant ($1.987\times 10^{-3}$ $\text{kcal}\cdot {\mathrm{mol}}^{-1}\cdot K^{-1}$), and *T* is the simulation temperature (303.15 K). According to transition state theory, *A* is constrained by the system’s internal friction, reflecting the intrinsic characteristics of its conformational dynamics [30,31]. Given that the global topology of the complex remains robust under THz excitation, the potential fluctuations in *A* have a negligible impact compared to the exponentially amplified kinetic effects driven by energy barrier (*F*) changes and can thus be safely neglected in our physical assessment. To intuitively compare the orders-of-magnitude acceleration effect of the resonant THz field on dissociation kinetics, we defined the logarithm of the relative dissociation constant ($\ln k_{\mathrm{rel}}$) as the ratio of the difference between the energy barriers of the control group ($F_{\text{ctrl}}$) and the THz-excited group ($F_{\exp}$) to the thermal energy:

$$\ln k_{\mathrm{rel}}=\ln\left(\frac{k_{\exp}}{k_{\text{ctrl}}}\right)=\frac{F_{\text{ctrl}}-F_{\exp}}{\mathrm{RT}}$$ (2)

Calculation results (Fig. 4C) indicate that the introduction of targeted THz field substantially enhanced the dissociation tendency of the complex. Under modulation at 36.6 and 43.0 THz, the $\ln k_{\mathrm{rel}}$ values reached 15.15 and 20.22, respectively, indicating a marked increase in dissociation rates. Most strikingly, the $\ln k_{\mathrm{rel}}$ for the 40.2-THz group surged to 44.37. This dramatic increase not only reaffirms the specificity of this frequency as an optimal modulation window but also confirms from a physicochemical perspective that resonant THz stimulation drastically lowers the activation energy via a nonthermal resonance process, thereby driving the complex to cross the thermodynamic barrier at an exponentially increased rate, achieving rapid and controllable dissociation of the binding interface. To further contextualize the physical feasibility of this process, we quantitatively estimated the absolute time scale of dissociation based on the established Arrhenius model. In characterizing the dissociation kinetics, the pre-exponential factor *A* is governed by the system’s intrinsic dynamics. According to theoretical derivations of rate constants from free energy profiles, the theoretical upper limit for microscopic local dissociation processes approaches the universal attempt frequency of $A\approx 10^{12}\;s^{-1}$ [32]. Substituting the barrier height under 40.2-THz excitation ($F_{\exp}=15.23$ kcal/mol) into Eq. (1) yields an estimated rate of $k\approx 10\;s^{-1}$. This corresponds to a characteristic decoupling time (τ=1/k) of approximately 0.1 s (100 ms). Compared to the practically negligible dissociation rate of the native state ($F_{\text{ctrl}}=41.94$ kcal/mol), this substantial kinetic acceleration successfully brings the dissociation process into a biologically accessible time scale.

To deeply investigate the microscopic structural basis of this dynamic process, we monitored the real-time conformational evolution of the complex in simulation trajectories (Fig. S13). Trajectory analysis showed that under the influence of a continuous THz field, the root-mean-square deviation climbed markedly and ultimately converged, while the radius of gyration ($R_{g}$) remained overall compact. This suggests that long-term THz irradiation does not cause destructive global unfolding or chaotic structural degradation of the protein but rather drives an internal structural phase transition that settles into a stable, “loosened” equilibrium state at the interface while maintaining the fundamental folding topology. To further elucidate the distribution characteristics of this local rearrangement in conformational space, we constructed the free energy landscape of the system using root-mean-square deviation and $R_{g}$ as reaction coordinates (Fig. 4D to G), where the color gradient directly maps the free energy (G) of the system. The control group (Fig. 4D) remained within a single, deep, and compact energy basin, indicating it resides in a low-entropy native rigid conformation. In contrast, the applied THz field induced varying degrees of energy landscape remodeling. The 36.6- and 43.0-THz groups (Fig. 4E and G) exhibited energy valley dispersion, indicating that these frequencies forced the complex to deviate from the native state and migrate toward higher-energy sparse regions. Notably, the 40.2-THz group displayed the most drastic conformational remodeling features (Fig. 4F). The conformational sampling range expanded substantially, and the originally dense native low-energy basin evolved into a multicentered topologically discrete state, implying that the complex was effectively trapped in non-native sparse states. Due to substantial energy barriers between the sparse and native states, the system struggles to spontaneously revert to the original bound state under continuous excitation. In summary, THz light remodels the thermodynamic stability of the binding interface by driving the system toward a high-entropy, disordered conformational ensemble, thereby inducing a directed phase transition of the interfacial structure.

### Validation of the interfacial sparsification effect

**Fig. 5.**
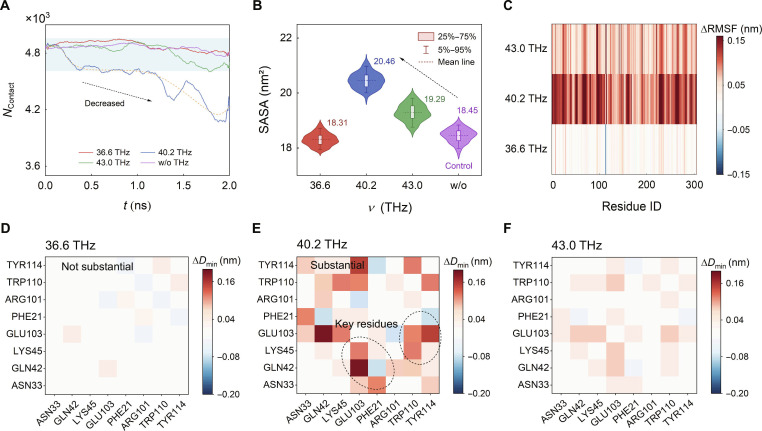
Validation of the dynamic responses and underlying processes of the PD-1/PD-L2 complex under terahertz (THz) light. (A) Spatiotemporal evolution of the number of contacts between key residues. A contact is defined as an inter-residue distance of less than 0.6 nm. (B) Changes in the solvent-accessible surface area (SASA) of the complex. (C) Difference in root-mean-square fluctuation (ΔRMSF) of all residues relative to the control group. (D to F) Heatmaps of the difference in the average minimum distance $\left(\Delta D_{\min}\right)$ between residues relative to the control group.

To systematically validate the physical basis underlying the aforementioned energy landscape remodeling and structural evolution from a full time-domain dynamic dimension, we further investigated the multilevel response characteristics of the PD-1/PD-L2 interface, ranging from the macroscopic topological network to microscopic core residues. First, we intuitively assessed the stability of interfacial connectivity by monitoring changes in the number of physical contacts ($N_{\text{contact}}$) between residues (Fig. 5A). Because the identification of each physical contact is fundamentally governed by a strict spatial geometric cutoff distance ($d_{\mathrm{cut}}=0.6$ nm), the temporal decay of this metric characterizes the spatiotemporal sparsification of the interfacial topological network. Dynamic analysis results indicate that the maintenance of these microscopic physical tethers exhibits starkly contrasting stability characteristics: In the control, 36.6-THz, and 43.0-THz groups, the number of contacts between key residues remained basically stable throughout the simulation period, demonstrating robust topological maintenance capability. In stark contrast, under the drive of the 40.2-THz excitation, the interfacial contact network underwent specific large-scale sparsification, with the number of contacts substantially decaying over time. This phenomenon marks that the complex interface has crossed a critical state, achieving a directional transition from a tight physically anchored state to a contact-relaxed state.

To verify whether this restructuring of the topological network triggered a qualitative change in interfacial physicochemical properties, we further quantified the global evolution of the SASA (Fig. 5B). Statistical results show that the impact of the applied THz field on interfacial exposure exhibits high-frequency sensitivity: The value for the 36.6-THz group (18.31 nm^2^) was comparable to the control group (18.45 nm^2^), while the 43.0-THz group induced only a slight numerical increase (19.29 nm^2^), indicating that interfacial compactness was well maintained at these 2 frequencies. Conversely, the 40.2-THz group showed a substantial increase to 20.46 nm^2^. This localized 10% SASA expansion induced by the specific resonant frequency mechanistically reflects the deshielding of the interfacial hydrophobic core: The structural phase transition exposes partial nonpolar residues, and water permeation loosens the tight interfacial packing. Comparison with the entire complex (Fig. S14) reveals that this limited area increase exhibits spatial anisotropy rather than global thermal fluctuation, proving a directed transition toward a porous interfacial state. Furthermore, this minor variation magnitude rules out macroscopic thermal denaturation, confirming the integrity of the native folded backbone.

Having completed the global topological validation, we further narrowed our focus to the dynamic details at the atomic scale. The difference in root-mean-square fluctuation (ΔRMSF) heatmap (Fig. 5C) reveals that the induction of protein flexibility by the incident THz field possesses high spatial heterogeneity and energy level matching characteristics. Statistics show that the 36.6- and 43.0-THz bands are lighter in color, indicating minimal difference in their flexibility profiles compared to the control group, inducing only weak fluctuations. In contrast, under 40.2-THz EM excitation, the bands largely appear deep red, indicating that the most intense conformational fluctuations were excited across the entire residue sequence range. These excited local degrees of freedom altered the originally precise geometric complementarity between receptor and ligand, constituting the key kinetic driving force for the complex’s transition to a loose state.

Finally, to quantitatively characterize the degree of physical uncoupling of the complex on a spatial scale, we calculated the difference in the average minimum distance ($\Delta D_{\min}$) between key interfacial residues (Fig. 5D to F). Results confirm that THz field-induced interfacial expansion exhibits strong frequency specificity, which is intuitively mapped by the distinct color gradients across the heatmaps. Under the action of the 36.6-THz field (Fig. 5D), no significant shift was observed in the physical distances between core residues ($\Delta D_{\min}<0.08$ nm); this insubstantial change is visually characterized by a clean, predominantly white or faint color tone, indicating the maintenance of the original compact binding state. Similarly, 43.0 THz induced only small-scale and low-intensity structural relaxation, manifesting as faded and localized patches (Fig. 5F). In sharp contrast, 40.2-THz excitation triggers a dramatic chromatic shift toward dense, dark red regions (Fig. 5E). This prominent color transition corresponds to a marked increase in the physical distance of interfacial core residue pairs ($\Delta D_{\min}>0.08$ nm), especially between GLU103 and GLN42/TYR114, signifying a profound macroscopic transition to a sparse state. These deep-red regions of high positive values intuitively depict the degree of this large-scale spatial reorganization at the binding interface. This quantitative analysis at the spatial scale directly confirms that the 40.2-THz field, by enhancing specific interfacial conformational plasticity, triggered the sparsification of the atomic contact network and subsequent hydrophobic deshielding, thereby achieving a precise interfacial decoupling of the PD-1/PD-L2 interface at the physical level through THz light.

Furthermore, to experimentally validate our computational findings, an enzyme-linked immunosorbent assay was employed to evaluate the impact of THz treatments at varying frequencies and power densities on the binding affinity between PD-1 and PD-L2. The results (Fig. S15) demonstrated a significant attenuation of the PD-1/PD-L2 binding capacity in the THz-irradiated groups, providing preliminary experimental corroboration of our simulation outcomes. Detailed experimental procedures and results are provided in the “Supplementary Experimental Validation” section of the Supplementary Materials.

## Conclusion

This study establishes the strategic feasibility of THz light as a reversible, nonthermal, and noninvasive physical stimulus. Utilizing MD simulations of the PD-1/PD-L2 complex as a theoretical model, we demonstrate that the applied THz field effectively induces spatiotemporal atomic rearrangement at the interfacial connections. This process drives the interface from a stable dense state to a sparse state, successfully achieving a directed structural phase transition at the biomolecular interface. From an energy analysis perspective, under the influence of THz excitation at 36.6, 40.2, and 43.0 THz, the binding energy (ΔG) shifts from −74.32 kcal/mol to −64.27, −22.45, and −60.00 kcal/mol, respectively. Simultaneously, the PMF barrier steeply attenuates from 41.94 kcal/mol down to 32.83, 15.23, and 30.38 kcal/mol. This directly reflects that the incident THz field can induce rearrangement at the PD-1/PD-L2 interface, thereby substantially reducing the interfacial binding affinity. At the microstructural level, the resonant EM field further sparsifies the topological contact network. Across these 3 frequencies, the average number of interfacial hydrogen bonds drops from 12.59 to 11.45, 4.10, and 7.22, while the average bond length elongates from 2.87 Å to 2.93, 2.97, and 2.95 Å, respectively. Ultimately, comparative analysis of THz field frequencies reveals that 40.2 THz represents the optimal resonance frequency for triggering the maximal interfacial structural phase transition, whereas the effects at 36.6 and 43.0 THz are relatively moderate. This marked distinction highlights the high-frequency selectivity of this physical modulation.

Although this study utilized the PD-1/PD-L2 complex as a representative model with clear pharmacological relevance, the findings possess universal biophysical implications extending beyond this specific model. The multidimensional physical analysis paradigm established here, integrating spectral signatures, energy landscapes, and dynamic evolution, clearly elucidates the physical mechanism of the THz light-driven interfacial structural phase transition. Specifically, resonant excitation drives a nonequilibrium conformational rearrangement that effectively alters the steady state and triggers a cascade of events involving the solvation exposure of the hydrophobic core and the relaxation of the contact topological network, ultimately leading to the sparsification of the binding interface. Importantly, this process preserves the intact native folded backbone and relies primarily on the dynamic reconfiguration of noncovalent bonds. Consequently, the complex is merely driven into a metastable sparse state, thermodynamically possessing the full potential to revert to its bound conformation upon the removal of the external field. This theoretical insight offers a novel physical modulation strategy. It contributes to understanding how THz light functions as a noninvasive regulator to manipulate the steady-state evolution of complex biological macromolecular assemblies. We anticipate that these theoretical findings, grounded in the structural phase transition process of the biological macromolecular interface, will provide theoretical support for further in-depth experimental validations.

## Materials and Methods

### Model construction and parameterization

The PD-1/PD-L2 complex was centered within a cubic simulation box with dimensions of 21 × 21 × 21 nm^3^. The system was solvated using the TIP3P [33] water model (comprising a total of 290,200 water molecules) and neutralized by the addition of 830 chloride (${\mathrm{Cl}}^{-}$) and 835 sodium (${\mathrm{Na}}^{+}$) ions, thereby establishing a physiological salt concentration of approximately 0.15 M (Fig. S1). All-atom MD simulations were performed utilizing the CHARMM36m force field [34] with periodic boundary conditions applied in all 3 dimensions. Following energy minimization, the system was effectively equilibrated for 100 ns at 303.15 K and 1 bar using GROMACS (Fig. S2).

In the framework of classical MD simulations, since the velocity of charged particles in biological systems is negligible compared to the speed of light, the magnetic Lorentz force can be completely ignored relative to the electric field force [35]. Therefore, the external THz field can be equivalently modeled as a spatially uniform, time-varying classical electric field [36] $E\left(t\right)$, expressed as the following equation [37]:

$$E\left(t\right)=E_{0}u\text{cos}\left(\omega t+\phi \right)$$ (3)

where $E_{0}$ represents the electric field amplitude (set to 1.0 V/nm), u is the unit polarization vector (e.g., $u=\left(0, 0, 1\right)$ for the *z*-axis), and ϕ=0 is the initial phase. An intensity on the order of 1.0 V/nm has been proven to be sensitive and effective for biological macromolecules, allowing for the explicit capture of interfacial THz responses such as hydrogen bond sparsification, residue vibrational modes, and conformational remodeling without being overshadowed by background thermal noise [38–40]. The angular frequency ω is related to the excitation frequency ν by the equation ω=2πν.

### Calculation of absorption spectra

THz radiation absorption spectra were obtained via the Fourier transform of the dipole moment derivative autocorrelation function [41]. Following system equilibration, spectral sampling was performed with a 1-fs resolution over a total trajectory length of 50 ps. First, partial charges and instantaneous velocities of atoms within specific residue regions were extracted to compute the total dipole moment derivative, defined as:

$$\overset{\cdot }{M}\left(t\right)=\sum_{i=1}^{N}q_{i}v_{i}\left(t\right)$$ (4)

where *N* denotes the total number of atoms in the selected region and $q_{i}$ and $v_{i}\left(t\right)$ represent the partial charge and instantaneous velocity vector of the *i*-th atom at time *t*, respectively. To eliminate dynamic fluctuations during the initial evolution phase and ensure rigorous statistical ensemble equilibration, the first 40 ps of the sampling trajectory was discarded. Only the strictly stationary phase was extracted for subsequent analysis. Subsequently, the time autocorrelation function of this dipole current was calculated:

$$C\left(t\right)=\langle \overset{\cdot }{M}\left(0\right)\cdot \overset{\cdot }{M}\left(t\right)\rangle$$ (5)

Finally, a fast Fourier transform was applied to the normalized autocorrelation function, converting the time-domain signal into a frequency-domain power spectral density. This procedure yielded the characteristic THz absorption spectra for the specified regions of the protein interface.

### Calculation of the binding energy

The binding energy (ΔG) between PD-1 and PD-L2 was rigorously quantified using the molecular mechanics/generalized Born surface area method [42], implemented via the GROMACS-compatible gmx_MMPBSA toolkit. To ensure statistical reliability, the final binding free energies were calculated by averaging over 51 frames extracted from the equilibrium phase of the MD trajectories. The total binding energy of the system was decomposed into 4 distinct energetic contributions, expressed as:

$$\Delta G=\Delta_{\mathrm{VDW}}+\Delta_{\mathrm{EEL}}+\Delta_{\mathrm{EGB}}+\Delta_{\text{ESURF}}$$ (6)

Here, $\Delta_{\mathrm{VDW}}$ and $\Delta_{\mathrm{EEL}}$ denote the van der Waals and electrostatic interaction energies in vacuum, respectively. The solvation free energy comprises 2 components: The polar solvation free energy ($\Delta_{\mathrm{EGB}}$), derived using the Generalized Born (GB) model, and the nonpolar solvation free energy ($\Delta_{\text{ESURF}}$), estimated based on the SASA utilizing the LCPO (linear combination of pairwise overlaps) algorithm.

### Calculation of the PMF

To quantify the free energy barrier and construct the PMF profile, umbrella sampling simulations were performed [43]. Initially, the ligand was pulled away from the receptor along the *z*-axis at a constant velocity of 10 nm/ns using a harmonic potential with a force constant of 1,000 $\mathrm{kJ}\cdot {\mathrm{mol}}^{-1}\cdot {\mathrm{nm}}^{-2}$. This nonequilibrium pulling process generated a continuous dissociation trajectory from the native bound state to the fully dissociated state.

Subsequently, a series of overlapping sampling windows were extracted along the reaction coordinate. Within each independent window, the system underwent 5 ns of equilibration followed by a 2-ns production run to collect conformational data. A harmonic restraining potential was applied to maintain the system within the specific phase space of each window. Finally, the weighted histogram analysis method was employed to integrate the distributions and reconstruct the continuous PMF curve by eliminating the influence of biasing potentials.

## Data Availability

All data necessary to understand and assess the conclusions of this study are available in the main text or the Supplementary Materials. There are no restrictions on data availability in the manuscript.

## Supplementary Materials

Supplementary 1 Figs. S1 to S15 References [44–52]
