# Supplementary material for: Computational Study of Terahertz-Induced Phase Transitions at Molecular Interfaces
Source: Research (Wash D C). 2026 Jul 28;9:1350. doi: 10.34133/research.1350 (PMC13408037; doi:10.34133/research.1350)
Supplement: Supplementary 1 — Figs. S1 to S15 References [44–52] [file research.1350.f1.docx]

*Supplementary Material*

**Computational Study of Terahertz-induced Phase Transitions**

**at Molecular Interfaces**

Chenzhi Tang^1†^, Dexing Shen^1†^, Zhi Du^1†^, Yong He^2^, Yingying Sun^1^, Ze Wang^1^, Yuanyuan He^3*^, Xingang Ren^1,4*^, and Kaijie Wu^1,4*^

^1^School of Electronic and Information Engineering, Anhui University, Hefei 230601, China.

^2^School of Electronics, Peking University, Beijing 100871, China.

^3^School of Safety Engineering, University of Emergency Management, Hebei 065201, China.

^4^Information Materials and Intelligent Sensing Laboratory of Anhui Province, Anhui University, Hefei 230601, China.

^*^Address correspondence to: heyy21@pku.edu.cn (Yuanyuan He); xgren@ahu.edu.cn (Xingang Ren); 23109@ahu.edu.cn (Kaijie Wu)

^†^These authors contributed equally to this work.

1. **Supplementary Figures**


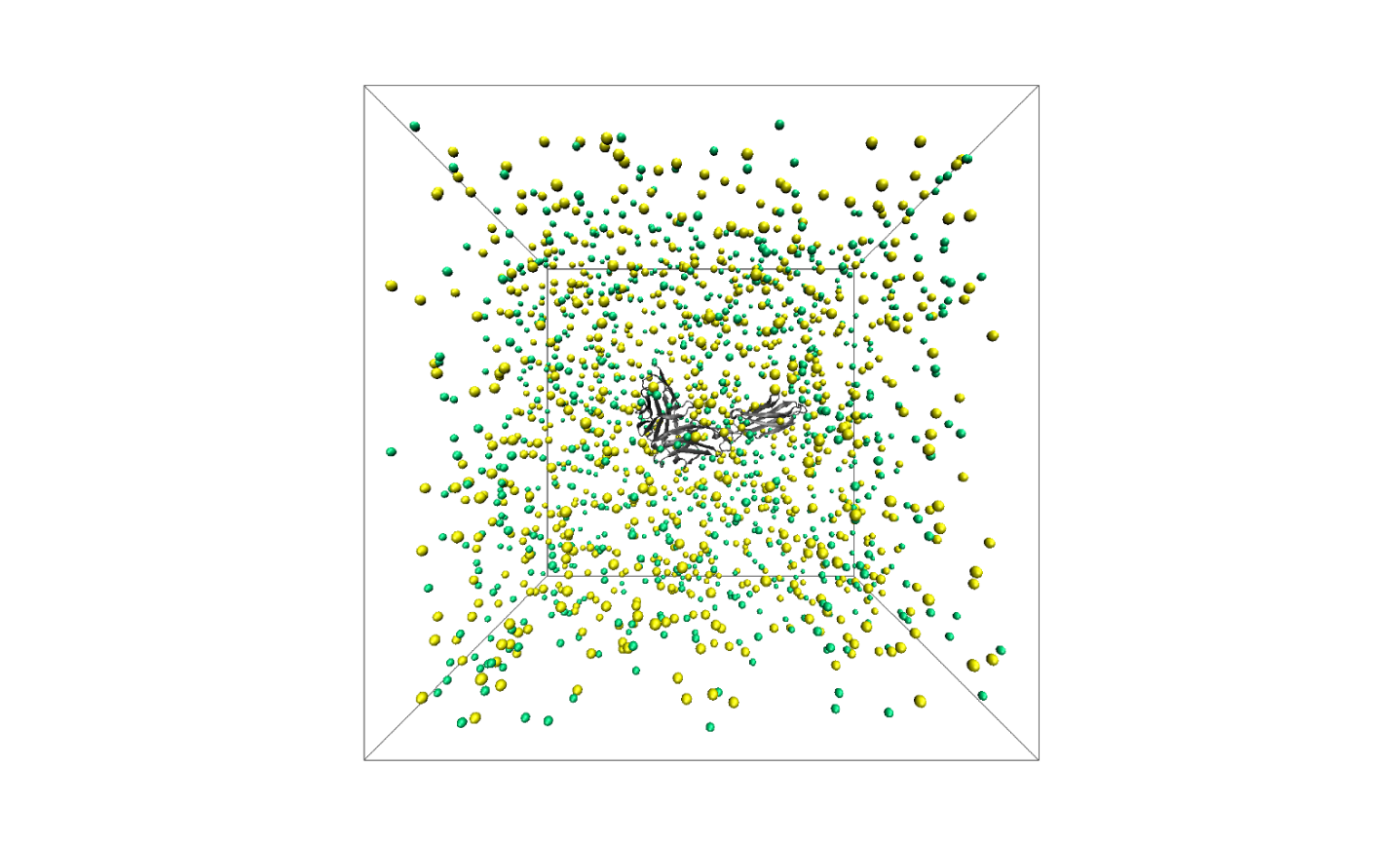


**Figure S1.** Initial structural conformation established for molecular dynamics simulations. The physical boundaries of the cubic simulation box are demarcated by the black frame. The 835 sodium ions and 830 chloride ions added to the system are depicted as green and yellow spheres respectively. To ensure optimal visualization of the protein backbone topology and the spatial distribution of ions, the 290,200 solvent water molecules within the system are omitted from this rendering.


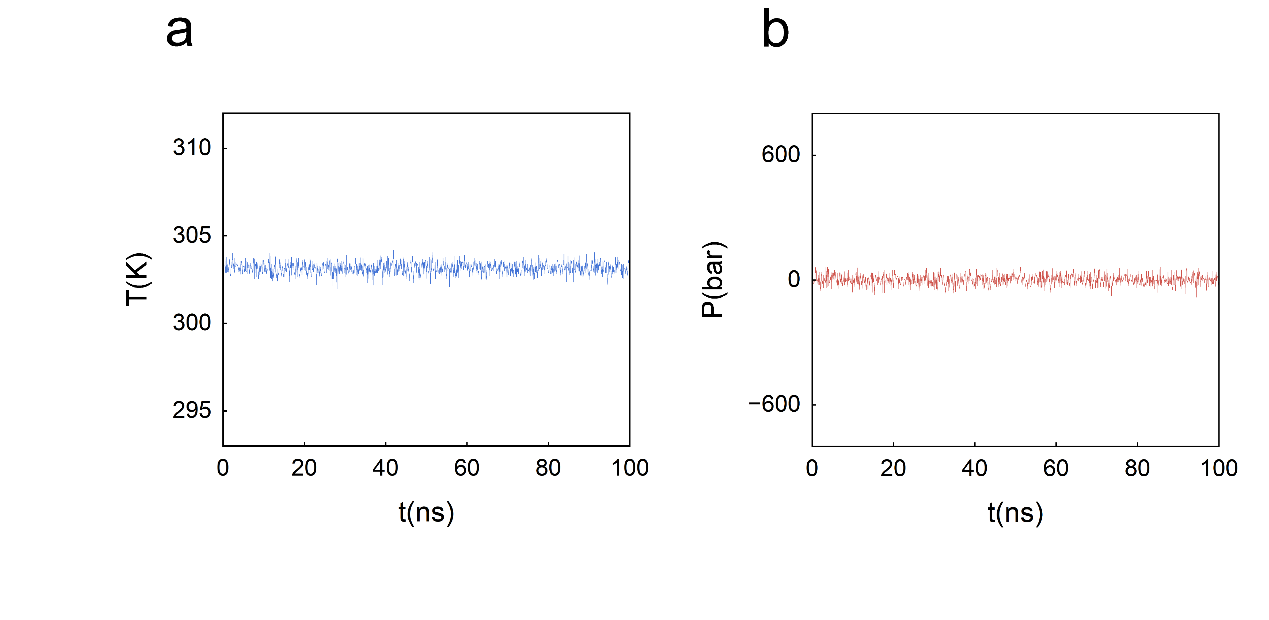


**Figure S2.** Thermodynamic equilibration profiles of the simulation system. (a) Temporal evolution of the internal temperature during the equilibration phase. (b) Temporal fluctuations of the macroscopic pressure across the equilibration trajectory.


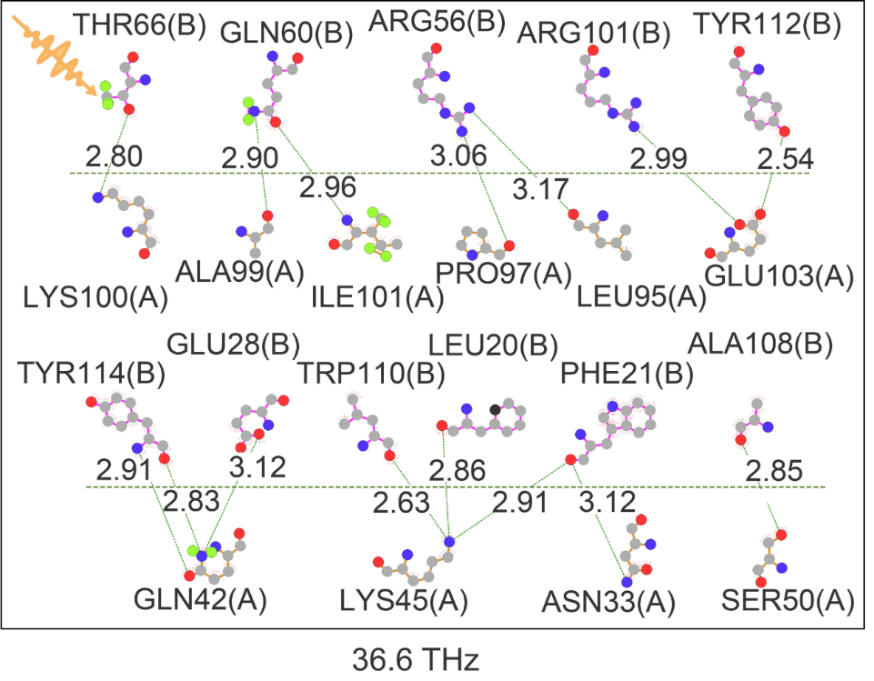


**Figure S3.** Visualization of the topological evolution of the interfacial hydrogen bond network under 36.6 THz EM field modulation. Atoms are colored as follows: carbon (gray), nitrogen (blue), oxygen (red) and other atoms (green). Green lines denote H-bonds connections between residues.





**Figure S4.** Calculated terahertz absorption spectra of the PD-1/PD-L2 complex and water solvent derived from molecular dynamics simulations. The spectrum over the frequency domain ($\nu$) is categorized into four distinct regions based on interaction characteristics: the low-absorption band (red shading), the water absorption bands (blue shading) where substantial solvent energy shielding occurs, the effective resonance window spanning 32-44 THz (green shading), and the localized covalent vibration band at higher frequencies (yellow shading).


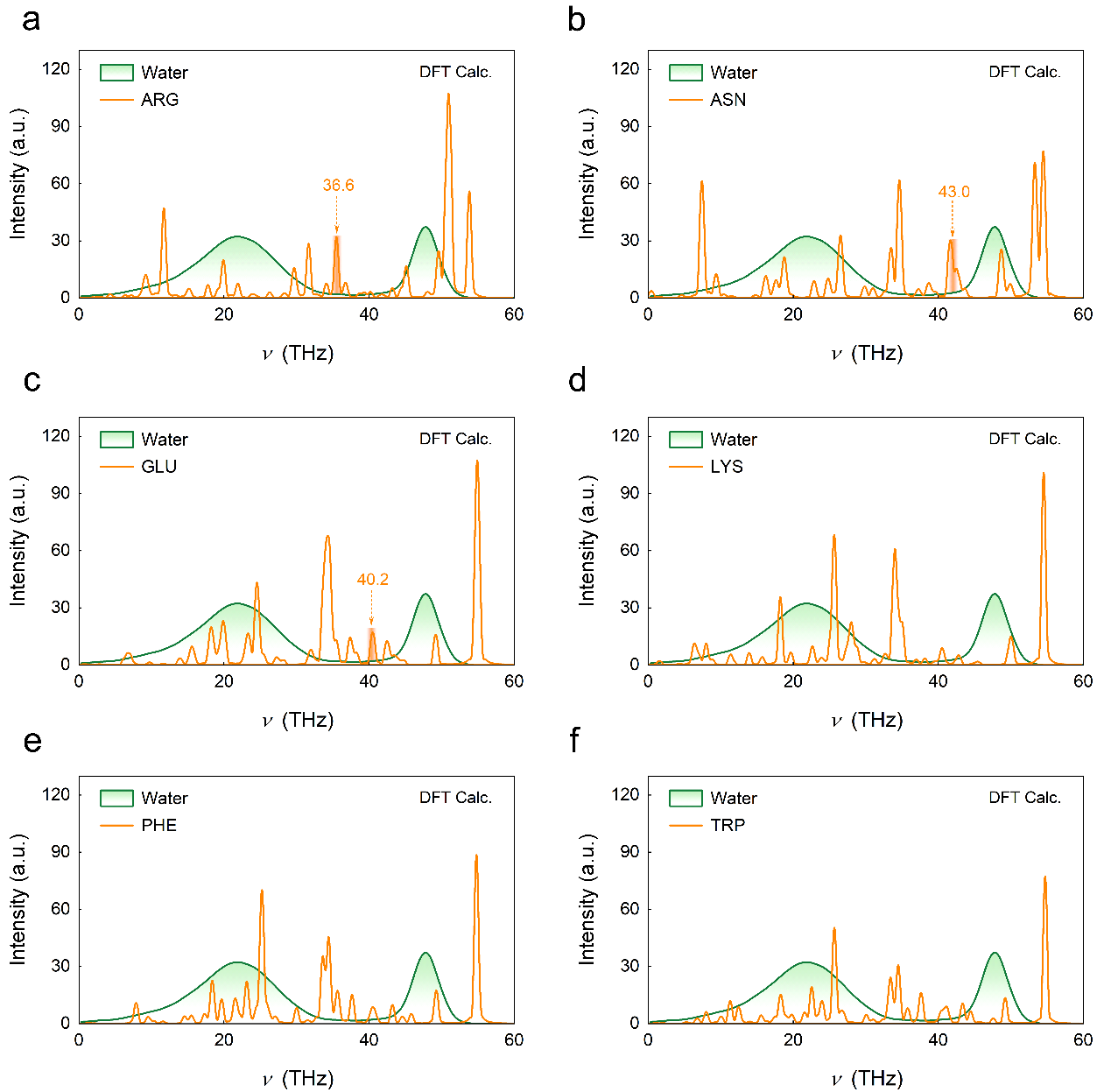


**Figure S5.** DFT calculated absorption spectra of Arginine (ARG), Asparagine (ASN), Glutamic acid (GLU), Lysine (LYS), Phenylalanine (PHE) and Tryptophan (TRP).





**Figure S6.** Comparative analysis of intermolecular binding energy ($\Delta G$) under specific single-residue resonance excitations. The bar chart illustrates the binding energy variations when targeting the isolated resonance peaks of individual hotspot residues (ARG corresponding to 32.0 THz, GLU to 34.5 and 38.0 THz, and TYR to 36.6 THz) compared to the collective resonance mode at 40.2 THz and the unperturbed control group (w/o).


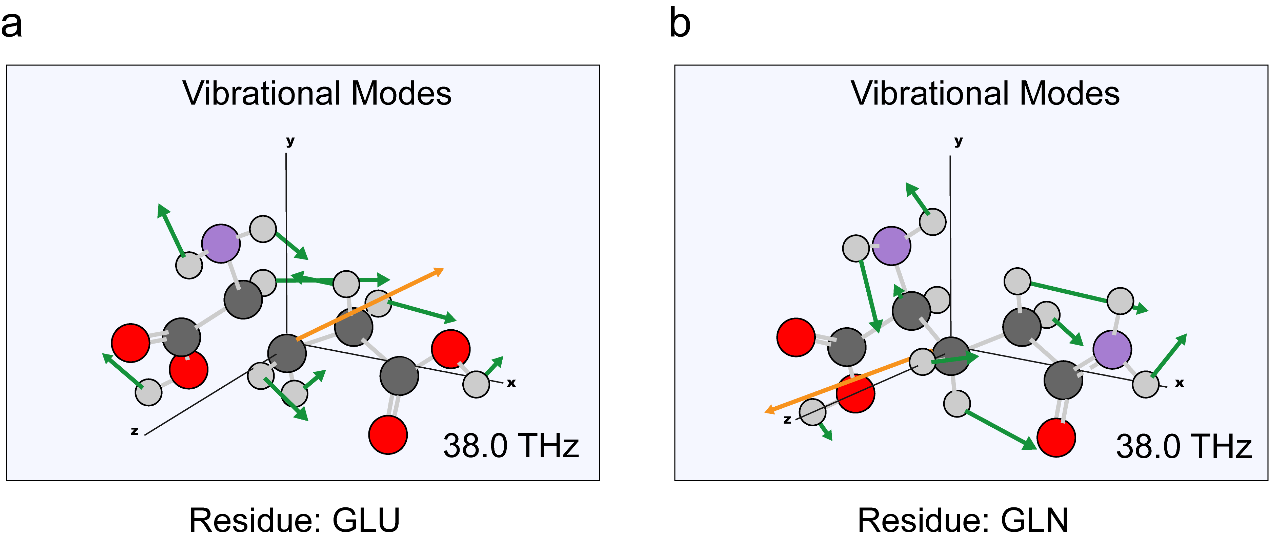


**Figure S7.** Visualization of the opposing vibrational modes of GLU and GLN residues under 38.0 THz excitation. (a) and (b) illustrate the localized structural motions of GLU and GLN, respectively.


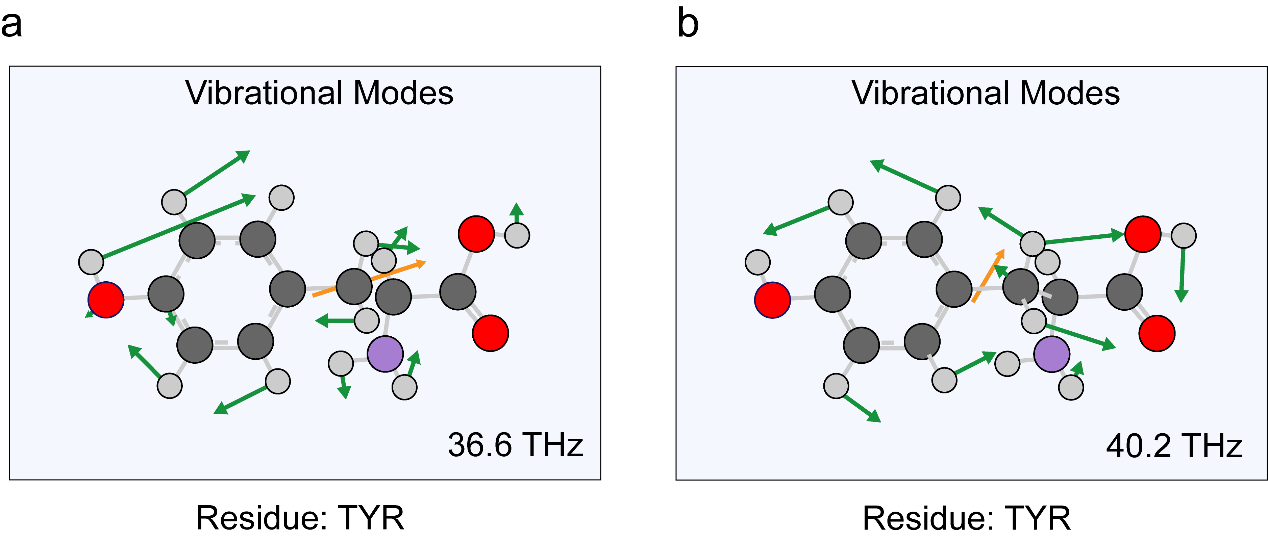


**Figure S8.** Vibrational modes of the TYR114 residue at 36.6 THz (resonant) and 40.2 THz (non-resonant). At 36.6 THz, synergistic side-chain stretching generates substantial dipole moment fluctuations. In contrast, at 40.2 THz, conflicting atomic displacement vectors neutralize the overall dipole moment change, resulting in the observed baseline absorption.





**Figure S9.** Changes in the global solvent-accessible surface area (SASA) of the PD-1/PD-L2 complex under different THz electric field intensities.





**Figure S10.** Probability distributions of interfacial H-bond lengths within the PD-1/PD-L2 complex under varying THz EM stimuli. The statistical profiles are derived from the ensemble analysis of the entire molecular dynamics trajectories. The vertical axis represents the relative frequency (probability, integrating to 100%), which provides an intrinsic statistical normalization of the data. Unlike isolated normal mode atomic displacements, this normalized ensemble distribution directly reflects the dynamic structural fluctuations of the highly polar non-covalent network, effectively serving as the microscopic physical carrier of the dipole moment responses.





**Figure S11.** Changes in the N-O atomic distances of key hydrogen-bond forming residues.


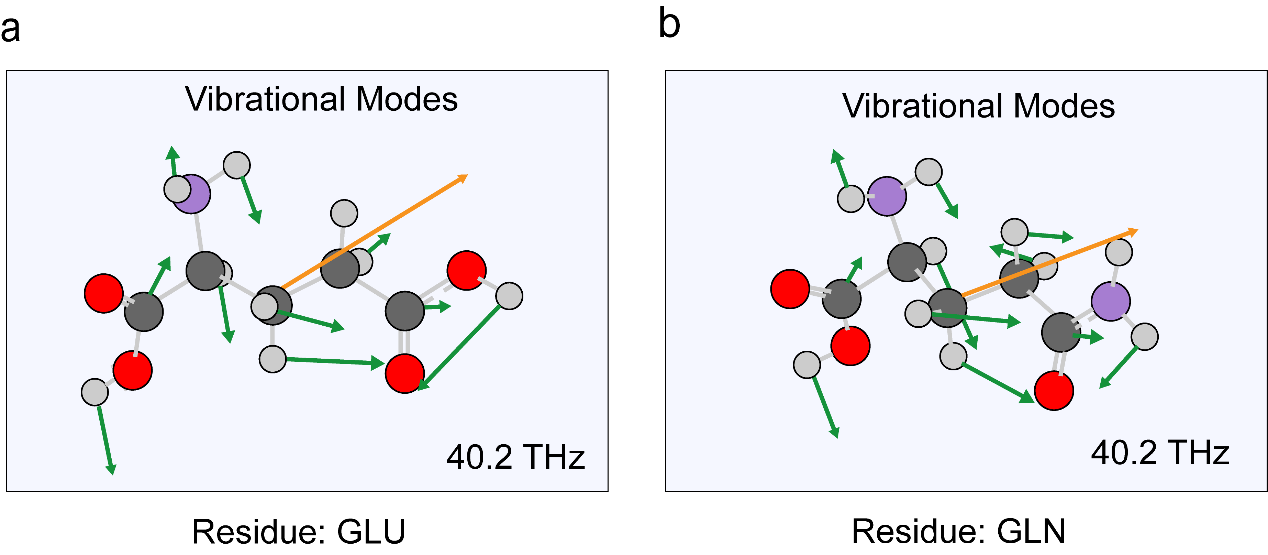


**Figure S12.** Normal-mode eigenvector analysis of interfacial GLU (a) and GLN(b) residues under 40.2 THz stimulus.


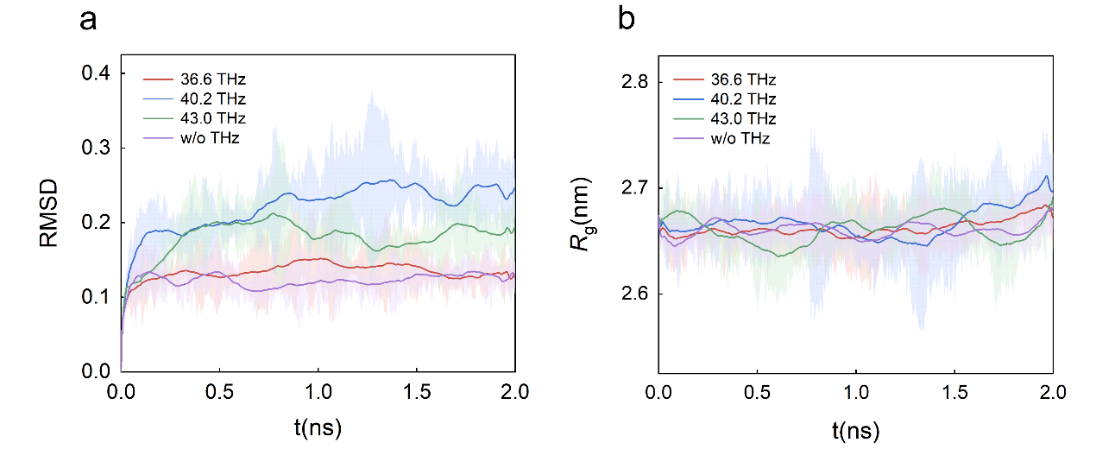


**Figure S13.** Structural dynamic evolution of the PD-1/PD-L2 complex modulated by THz EM field at varying frequencies. (a) Temporal evolution of the backbone root mean square deviation (RMSD). (b) Temporal evolution of the radius of gyration (R_g_). For both panels, the solid lines represent the mean values derived from three independent replicate simulations, and the surrounding shaded bands indicate the corresponding standard deviation (SD) at each time point.





**Figure S14.** Overall solvent-accessible surface area (SASA) of the PD-1/PD-L2 complex under varying THz irradiation frequencies.

1. **Supplementary Methods**
   1. **DFT calculations and vibrational modes**

To elucidate the molecular origins of the characteristic terahertz absorption peaks, key interfacial residues identified in the MD simulations were extracted to construct independent capped models. DFT calculations were subsequently performed [44] utilizing the Gaussian software.

Following rigorous full geometry optimization and frequency analysis of each residue model, the calculated vibrational frequencies were subjected to appropriate scaling factors and spectral broadening to generate theoretical absorption spectra for comparative validation. Finally, detailed vibrational assignments of the normal modes were performed using the GaussView visualization software. This facilitated a direct correlation between the macroscopic absorption peaks and the intrinsic vibrational modes of specific side-chain groups, thereby unambiguously confirming the physical basis of the resonant coupling between the terahertz field and the protein at the quantum mechanical level.

**2.2 Calculation of the RMSD**

To quantitatively characterize the dynamic temporal evolution and the degree of conformational deviation of the complex driven by the optical field, the RMSD of the protein backbone atoms relative to the initial reference structure was calculated. To eliminate the overall translational and rotational motions of the system, each trajectory frame was superimposed onto the reference structure via least-squares fitting [45] prior to the calculation. The RMSD is mathematically defined as:

$$\text{RMSD}(t)=\sqrt{\frac{1}{N}\sum_{i=1}^{N} \left\|\mathbf{r}_{i}(t)-\mathbf{r}_{i}^{\text{ref}}\right\|^{2}}$$

where $N$ denotes the total number of selected atoms, and $\mathbf{r}_{i}(t)$ and $\mathbf{r}_{i}^{\text{ref}}$ represent the position vectors of the $i$-th atom at time $t$ and in the reference structure, respectively.

**2.3 Calculation of the *R*_g_**

The radius of gyration (*R*_g_) was calculated to characterize the folding compactness and overall shape variations of the protein [46]. Mathematically, *R*_g_ is defined as the mass-weighted root-mean-square distance of atomic positions relative to the center of mass of the system:

Here, $m_{i}$ represents the mass of the $i$-th atom, while $\mathbf{r}_{i}(t)$ and $\mathbf{r}_{\text{CM}}(t)$ denote the position vector of the $i$-th atom and the center-of-mass vector of the system at time $t$, respectively.

**2.4 Calculation of the free energy landscape**

To elucidate the conformational space and metastable state distribution of the PD-1/PD-L2 complex driven by the terahertz field, and to clarify the thermodynamic principles underlying its steady-state transition [47], the joint probability distribution was calculated by employing $\text{RMSD}$ and R_g_ as the two-dimensional reaction coordinates. Subsequently, the Gibbs free energy landscape of the system was reconstructed via the Boltzmann inversion relation:

$$G(R_{g},\text{RMSD})=-k_{B}T\ln\left[\frac{P(R_{g},\text{RMSD})}{P_{\text{max}}}\right]$$

Here, $k_{B}$ represents the Boltzmann constant, $T$ denotes the simulation temperature (303.15K), $P(x,y)$ is the probability distribution of the system state along the reaction coordinates $(x,y)$, and $P_{\text{max}}$ is the maximum probability corresponding to the global energy minimum (defined as $G=0$).

**2.5 Analysis of H-bonds characteristics**

The dynamic topological evolution of the protein-protein interfacial H-bonds network serves as a critical metric for evaluating the binding steady state of the complex. The spatiotemporal evolution characteristics of the H-bonds network density between the PD-1 and PD-L2 interfaces, driven by the terahertz field, were systematically quantified from the simulation trajectories. To ensure robust statistical significance, the temporal evolution of H-bonds populations was analyzed across 500 frames evenly sampled from the production trajectory. The determination of H-bonds strictly adhered to standard physical geometric criteria [48]: a hydrogen bond was assigned when the donor-acceptor (D-A) distance ($r_{\text{DA}}$) was $\leq0.35\text{nm}$, and the hydrogen-donor-acceptor angle ($\angle\text{H-D-A}$) was $\leq30^{^{\circ}}$.

To further quantify the physical linkage strength and the degree of conformational relaxation of specific key interface residue pairs at the atomic scale, the geometric lengths of interfacial H-bonds were precisely tracked. Specifically, 10 representative instantaneous conformations were uniformly extracted from the simulation trajectory. These conformations were visually analyzed utilizing LIGPLOT+ software, enabling the precise extraction and statistical evaluation of the bond length parameters for hydrogen bonds between core interfacial residues. This approach visually elucidates how specific physical perturbations induced by the applied THz field drive a directional sparsification phase transition within the interfacial contact network.

**2.6 Analysis of key interfacial residue contacts**

To quantify the dynamic binding tightness between key residues of PD-1 and PD-L2 at the atomic level, the temporal evolution of the number of interfacial contacts driven by the optical field was systematically tracked across the simulation trajectories. The contact number was calculated based on a distance threshold criterion, defined as the total number of atom pairs between the selected receptor and ligand residue groups within a distance of $\leq0.6\text{nm}$ in each trajectory frame [49]. The mathematical model of this metric, $N_{\text{contact}}(t)$, is defined as follows:

$$N_{\text{contact}}(t)=\sum_{i=1}^{N_{A}} \sum_{j=1}^{N_{B}} \Theta(d_{\text{cut}}-r_{ij}(t))$$

Here, $N_{A}$ and $N_{B}$ denote the total number of atoms in the two selected residue groups, respectively, and $r_{ij}(t)$ represents the Euclidean distance between atom $i$ and atom $j$ at time $t$. $\Theta(x)$ is the Heaviside step function, where $\Theta(x)=1$ if $x\geq0$, and $\Theta(x)=0$ otherwise. $d_{\text{cut}}$ is the defined cutoff radius ($0.6\text{nm}$). This quantitative spatial analysis precisely elucidates the local conformational rearrangements, the sparsification of physical linkages, and the directional evolution of topological stability at the binding interface over nanosecond timescales induced by the applied THz field.

**2.7 Calculation of the SASA**

The SASA is defined as the surface area of the locus traced by the center of a solvent probe sphere as it rolls continuously over the van der Waals surface of the solute. Topologically, the overall SASA of the complex is calculated as the spatial sum of the accessible surface areas of all constituent atoms. Its discretized mathematical formalism is expressed as:

$$\text{SASA}=\sum_{i=1}^{N} (R_{i}+r_{\text{probe}})^{2}\Omega_{i}$$

Here, $N$ denotes the total number of atoms within the target region, $R_{i}$ represents the van der Waals radius of the $i$-th atom, $r_{\text{probe}}$ is the effective probe radius simulating a water molecule (standardized to 0.14 nm in this study), and $\Omega_{i}$ defines the accessible solid angle of the atom that is not geometrically occluded by adjacent neighboring atoms. In this study, the aforementioned integral was numerically solved utilizing the double cubic lattice method proposed by Eisenhaber [50]. To accurately capture the dynamic topological exposure of the complex, the SASA was calculated across 500 frames uniformly sampled from the simulation trajectory.

**2.8 RMSF analysis**

To evaluate the impact of the terahertz field on the local structural flexibility of the PD-1/PD-L2 complex, the RMSF of the backbone atoms was calculated. RMSF serves as a robust metric to quantify the deviation of protein residues from their time-averaged positions [51], mathematically defined as:

$$\text{RMSF}_{i}=\sqrt{\frac{1}{T}\sum_{t=1}^{T} \left\|\mathbf{r}_{i}(t)-\langle\mathbf{r}_{i}\rangle\right\|^{2}}$$

Here, $T$ denotes the total number of sampled trajectory frames, $\mathbf{r}_{i}(t)$ represents the spatial position vector of the $i$-th residue at time $t$, and $\langle\mathbf{r}_{i}\rangle$ is the time-averaged position vector of that residue across the entire equilibrated trajectory. For these calculations, the backbone index group of the protein complex was selected, yielding an averaged RMSF value per residue.

To intuitively elucidate the specific modulatory effects of THz EM field excitation on local protein flexibility, a differential RMSF ($\Delta\text{RMSF}_{i}$) analysis was introduced. Specifically, the baseline RMSF values of the field-free control group ($\text{RMSF}_{i}^{\text{Control}}$) were subtracted from the corresponding RMSF values obtained under various terahertz field frequencies (36.6 THz, 40.2 THz, and 43.0 THz, denoted as $\text{RMSF}_{i}^{\text{THz}}$):

$$\Delta\text{RMSF}_{i}=\text{RMSF}_{i}^{\text{THz}}-\text{RMSF}_{i}^{\text{Control}}$$

A positive value ($\Delta\text{RMSF}>0$) indicates that the terahertz field enhances the flexibility of the respective region, whereas a negative value ($\Delta\text{RMSF}<0$) denotes increased rigidity. This high-resolution spatial differential analysis provides direct dynamical evidence for precisely pinpointing the core residues that undergo local conformational rearrangements and structural phase transitions driven by the external field.

**2.9 Key residues distance analysis**

To precisely quantify the physical perturbation effects of the terahertz field on the spatial topological arrangement of core residues at the PD-1/PD-L2 binding interface, the average minimum inter-residue distances of representative key interface residue pairs were first extracted from the equilibrated trajectories. Subsequently, to directly quantify the local spatial structural deviations induced by THz excitation, a difference distance profile ($\Delta D_{\text{m}}$) analysis was performed [52]. Specifically, the elements of the difference profile were obtained by subtracting the baseline average distance matrix of the field-free control system ($D_{ij}^{\text{Control}}$) from that of the system driven by a specific THz frequency ($D_{ij}^{\text{THz}}$), mathematically expressed as:

$$(\Delta D_{\text{m}})_{ij}=D_{ij}^{\text{THz}}-D_{ij}^{\text{Control}}$$

Within this reconstructed difference profile, positive values ($(\Delta D_{\text{m}})_{ij}>0$) intuitively indicate that the energy injection from the THz EM field induces an expansion and spatial stretching of the physical distance between residue $i$ and residue $j$. Conversely, negative values ($(\Delta D_{\text{m}})_{ij}<0$) denote a reduction in local spacing and spatial compaction. This selective spatial coordinate analysis provides quantitative geometric and topological evidence for deciphering the structural phase transitions at the complex interface during the steady state transition.

1. **Supplementary Experimental Validation**

**3.1 Materials**

The human Programmed Death-Ligand 2 (PD-L2) ELISA kit used in this study was purchased from MEIMIAN (Catalog No. MM-63896H1). Recombinant PD-L2 protein (Catalog No. ZYA63108Hu) and recombinant PD-1 protein (Catalog No. ZYA64627Hu) were obtained from Zeye Bio-Technology Co., Ltd., both with a guaranteed purity of ≥ 95%.

**3.2 THz light source**

A pulsed Quantum Cascade Laser (QCL) from Daylight Solutions Inc. was utilized to precisely control the emission wavelength and power. The experimental samples were exposed to THz waves at specific frequencies of 40.2 THz, 37.0 THz, and 32.0 THz, featuring a repetition rate of 200 kHz and a pulse width of 500 ns. The applied irradiation powers were approximately 3 mW and 9 mW.

**3.3 PD-L2 ELISA**

Prior to the THz exposure, recombinant PD-L2 and PD-1 proteins were mixed at a 1:1 ratio, and the complex concentration was adjusted to 10 ng/mL in the prepared samples. Subsequently, samples in the experimental groups were placed in the THz treatment setup and irradiated at the pre-defined frequencies and powers for 10 minutes, whereas the control group received no THz treatment.

For the ELISA detection, 50 μL of standards or samples (diluted 1:4) were added to a 96-well plate pre-coated with biotinylated anti-PD-L2 antibodies. Next, 100 μL of horseradish peroxidase (HRP)-conjugated streptavidin was added to each well, followed by incubation at room temperature for 1 hour. After discarding the solution from the wells, 300 μL of wash buffer was added to each well, and the plate was washed 5 times. Subsequently, 100 μL of substrate solution was added and incubated at 37 °C for 15 minutes in the dark. Finally, 50 μL of stop solution was added to each well and gently mixed. The absorbance values were immediately read at a wavelength of 450 nm.

**3.4 Statistical analysis**

All experimental data are presented as the mean ± standard deviation (SD). Statistical comparisons were performed using an unpaired t-test. A value of P < 0.05 was considered to indicate a statistically significant difference.

**3.5 Results**

Theoretically, a decrease in the binding affinity of the PD-1/PD-L2 complex would result in an increased generation of free PD-L2 in the solution. Therefore, the concentration of free PD-L2 directly serves as a reliable macroscopic indicator to reflect the intrinsic binding capacity of the complex. Accordingly, this study employed an ELISA to quantitatively evaluate the impact of THz treatments at varying frequencies and powers on the PD-1/PD-L2 binding affinity. The general experimental procedure is illustrated in Fig. S15a.

We recorded the differences in free PD-L2 concentration ($\Delta C_{\text{PD-L2}}$) between the THz-irradiated groups (at frequencies of 32.0, 37.0, and 40.2 THz, and powers of 3 mW and 9 mW) and the untreated control group. Experimental observations (Fig. S15b) revealed that the concentration of free PD-L2 in all THz-irradiated groups was significantly higher than that in the control group (P < 0.0001). This indicates that THz radiation across the tested parameters effectively attenuates the binding capacity between PD-1 and PD-L2, thereby providing preliminary experimental validation of our computational simulation results. Although the specific frequency-dependent effects exhibit a certain degree of deviation from the computational predictions, the overarching conclusion remains highly consistent. Given the inherent idealizations and simplified assumptions of the computational model, such discrepancies are reasonable. Subsequent experiments across additional dimensions will be conducted to further enhance the reliability of this theoretical study.


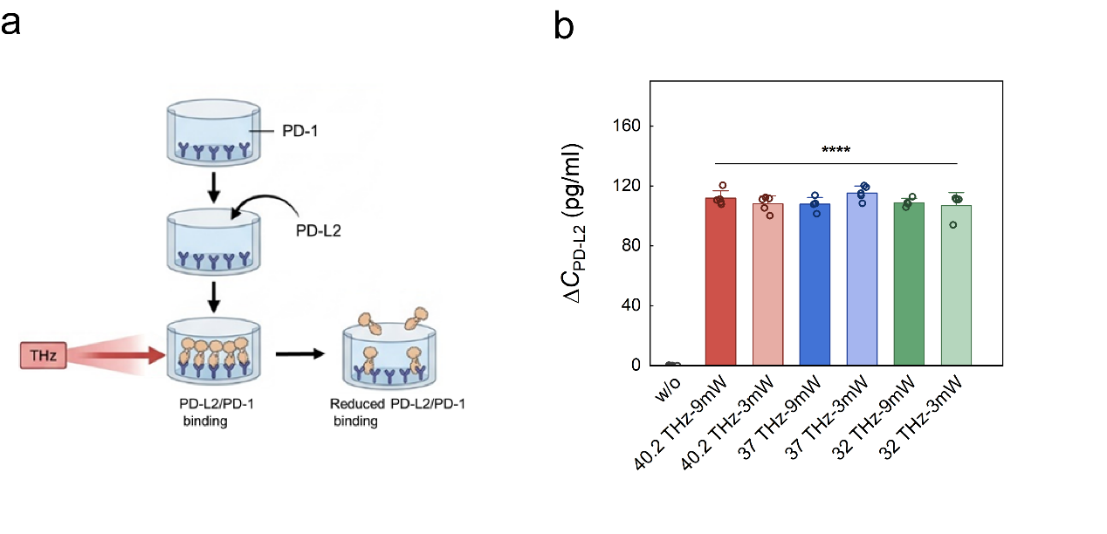


**Figure S15.** Experimental measurement of free PD-L2 concentration. (a) Schematic diagram of the experimental workflow. (b) Differences in free PD-L2 concentration relative to the control group under THz irradiation at varying frequencies and powers.
